# Relationship between cow parity and maternal parity on dairy cow lactation performance

**DOI:** 10.3168/jdsc.2025-0775

**Published:** 2025-05-22

**Authors:** D.P. Berry, K. Downing

**Affiliations:** 1 Teagasc, Animal & Grassland Research and Innovation Centre, Moorepark, Fermoy, Co. Cork, P61 P302, Ireland; 2 Irish Cattle Breeding Federation, Ballincollig, Co. Cork, P31 D452, Ireland

## Abstract

The effect of dam parity on the subsequent performance of her female progeny is of interest when exploring the cost-benefit of alternative herd-level breeding strategies. This is especially true with the growing adoption of sexed semen where proportionally more of the heifers and younger cows in a herd may be selected as candidate dams of the next generation. There is a paucity of information on the effect of, or association between, dam parity and subsequent progeny lactation productivity in dairy cows. The objective of the cross-sectional analysis of 1,833,875 cow lactation records in the present study was to quantify the association between dam parity (1 to 10+) and subsequent progeny lactation yield, milk fat and protein concentration, and SCC. Also of interest were model cow parity effects for these traits so as to inform cost-benefit analyses of improving cow lifespan. All associations were estimated using linear mixed models with cow included as a random effect while also facilitating heterogeneous dam and cow parity variance components. Mean yield increased with cow parity up to parity 5 (30% to 33% higher than yields from first parity cows) after which it declined; parity 10+ cows still produced, on average, 19% to 21% more milk than first parity cows although mean lactation SCC increased with advancing parity in multiparous cows. The mean 305-d lactation milk yield of cows increased as the parity number of their dams increased, peaking at parity 7 (19.8 kg more milk than progeny from first lactation dams) before declining. In contrast, peak fat and protein yields occurred at younger dam parities. Although the mean SCS of cows was greatest in progeny from parity 2 dams, mean lactation SCS declined thereafter as dam parity number increased. In conclusion, although dam parity was associated with progeny lactation performance, the biological extent of this association was almost negligible.

## Highlights

• Milk, fat, and protein yield increased with cow parity up to parity 5.
• Progeny from older dams yield more milk than younger, though the difference is small.
• Somatic cell count was highest in the progeny of first- and second-parity dams.

## Graphical Abstract

**Figure:**
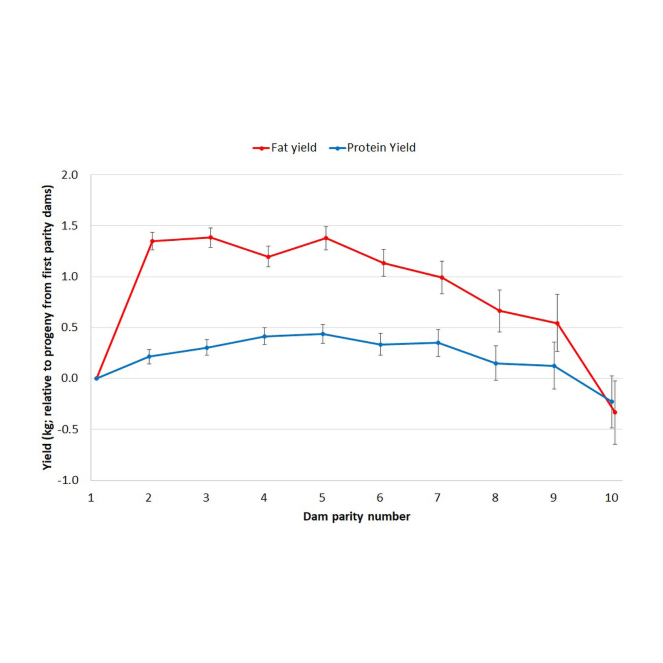
**Summary:** Given that younger animals are usually the most genetically superior cohort in a herd, the growing use of sexed semen often results in more herd replacements originating from younger dams. This study explored whether parity of dam was associated with the lactation performance of her progeny when they themselves become cows. While dam parity was associated with milk lactation performance of the progeny, the biological significance between different dam parities was relatively small, representing <0.6% of the yield of first-parity cows. Large cow parity effects on lactation performance were, however, detected with peak milk production at parity 5.

In dairy herds experiencing genetic improvement, the youngest animals are the most genetically elite cohort. Furthermore, irrespective of whether based on conventional or sexed semen, conception rates tend to be greatest in heifers (Maicas et al., 2019) relative to cow herd-mates. Primarily for these 2 reasons, nulliparous heifers are more likely to be chosen for mating with sex-sorted semen. This then begs the question whether the parity of the dam affects the lactation performance of the resulting female progeny. There is actually a paucity of recent studies in dairy cows that have explored the potential association between the parity of the dam and the subsequent lactation performance of her progeny (Storli et al., 2014; Van Eetvelde et al., 2020; Handcock et al., 2021). Furthermore, as reproductive performance in many dairy herds improve, an opportunity exists to increase the age profile in the herds. There is also a lack of recent estimates of parity effects on lactation performance of the cow herself, especially for older parity cows.

The objective therefore of the present study was to first explore the associations between the parity of the dam and the subsequent lactation performance of her progeny and second to estimate, using recent performance data, parity effects of the cow herself. Model solutions will be useful when quantifying the impact of various recommended mating schemes based on dam parity but also the impact of increasing cow lifespan.

Total lactation 305-d milk yield, fat yield, and protein yield were available from the Irish Cattle Breeding Federation national database for cows calving in the 5-yr period from 2020 to 2024; mean milk fat and protein percentage as well as arithmetic mean SCC were also available. Somatic cell count was transformed into SCS using the natural logarithm. The cows were predominantly Holstein-Friesian with some crossbreds, particularly between the Holstein-Friesian and Jersey. Genetic evaluations for the sire and dam of all cows from the Irish national genetic evaluation undertaken in February 2020 were also available. Genetic evaluations were available for all 3 yield traits, fat and protein percentage as well as SCS. The Irish national genetic evaluations for the 3 yield traits and, by extension, milk fat and protein percentage, are undertaken using an across-breed test-day model where parity 1, 2, and 3+ are treated as separate traits in a multitrait model. The genetic evaluations for each of the 3 yield traits are run separately. The predicted transmitting ability for the yield traits used in the present study is that which is published nationally, which is the weighted average of the individual parity predicted transmitting ability as 0.41:0.33:0.26 for parity 1, 2, and 3+, respectively. Breed is fitted via genetic groups in the founder population, which are allocated to breeds. The genetic evaluations are expressed across breeds. The genetic evaluations for SCS are undertaken using an animal repeatability model where log_e_SCC is the dependent variable. The estimated breeding value of all cows in the present study was simply the sum of the respective predicted transmitting ability of both parents; both the sire and dam of all cows had to have been recorded.

Only lactation data from cow parities 1 to 15 as well as those cows born from parity 1 to 15 dams were retained; due to a paucity of records in older parity animals, parities 10 and greater were recoded as a single group. Only performance data from cows that were producing in the same herd where they were born were retained. The median age at calving per parity was estimated for both the cow and the dam population separately, and lactations from calving ages more than 6 mo from the respective parity median were discarded. Age at calving relative to the parity median was subsequently categorized into months for later use in the statistical model. Heterosis and recombination loss coefficients were derived for all cows based on the breed composition of their respective parents (VanRaden and Sanders, 2003). Heterosis was categorized into 11 groups: 0%, >0% to ≤10%, 10% to ≤20%, …. >90% to ≤99%, and >99% to ≤100%. Recombination loss was categorized into 7 groups 0%, >0% to ≤10%, >10% to ≤20%, >20% to ≤30%, >30% to ≤40%, >40% to ≤50%, and >50%.

Information was also available on the extent of calving difficulty recorded for each calving. Calving difficulty in Ireland is scored by producers on a 4-point scale as (1) no assistance, (2) some assistance (assistance by one person), (3) considerable assistance (assistance by more than one person or assistance with a calf puller), and (4) veterinary assistance (including cesarean); a missing score was coded separately to facilitate the inclusion of this factor in the statistical model. Contemporary groups of herd-year-season of calving were derived using an approach routinely adopted for most of the national genetic evaluations in Ireland (Berry et al., 2013). Only contemporary groups with at least 10 records were considered further where the difference in calving date between the start and end of the contemporary group was no longer than 30 d. Following all edits, lactation performance data were available for 1,833,875 lactations from 814,485 cows producing across 70,303 contemporary groups in 6,341 herds. Of the 814,485 cows, 161,510 had >3.125% recorded Jersey ancestry and 93,490 had >3.125% recorded ancestry of other breeds. The mean recorded Holstein-Friesian proportion of the entire dataset was 0.91.

The association between lactation performance and both cow parity and dam parity was determined using a linear mixed model run in ASReml (Gilmour et al., 2009):

*Y_hijklmnop_* = CG*_h_* + Cow_parity*_j_* + Dam_parity*_k_* + Het*_l_* + Rec*_m_* + Cow_Age*_n_* + Dam_Age*_o_* + Dystocia*_p_* + b_1_EBV + b_2_JE + b_3_Other + Cow*_i_* + *e_hijklmnop_*,

where *Y_hijklmnop_* was the performance trait under investigation, CG*_h_* was the fixed effect of the contemporary group *h* (*h* = 70,303), Cow_parity*_j_* (*j* = 1 to 10+) was the parity of the cow herself, Dam_parity*_k_* (*k* = 1 to 10+) was the parity of the dam of the cow, Het*_l_* represented heterosis class *l* (*l* = 1 to 11), Rec*_m_* was the fixed effect of the recombination loss *m* (*m* = 1 to 7), Cow_Age*_n_* was the age of the cow, in months, relative to the median age for that respective parity in the cow population (n = 13), Dam_Age*_o_* was the age of the dam, in months, relative to the median age for that respective parity in the dam population (*o* = 13), Dystocia*_p_* was the recorded calving difficulty score for the parity under investigation (*p* = 5), EBV was the covariate for estimated breeding value with an associated regression coefficient b_1_, JE and Other were the covariates for the recorded breed proportion for JE and Other, respectively, with associated regression coefficients b_2_ and b_3_, respectively, Cow*_i_* was the random effect of cow where Cow*_i_* ∼iid $N\left(0,\sigma_{cow}^{2}\right),$ with $\sigma_{cow}^{2}$ denoting the cow variance, and *e_hijklmnop_* was the residual term where heterogeneous variances were fitted for both dam parity and cow parity.

The mean (median) parity of the dams in the study was 3.19 (3.00) with the number of records per dam parity in Table 1. The mean (median) parity of the cows in the study was 3.27 (3.00) with the number of records per parity in Table 2. The predicted marginal mean first parity milk, fat, and protein yield (averaged across all fixed effects) was 5,533, 240.3, and 199.8 kg, respectively; the predicted marginal mean first parity milk fat and protein concentration was 4.39% and 3.63%, respectively, whereas the mean SCS was 11.10 log_e_SCC units.

**Table 1:** Number of records (N), along with yields and composition (including SCS), relative to first parity dams (SE in parentheses), for cows born to dams of different parities

Parity	N	Yield (kg)			Composition (%)		SCS (log_e_ units)
		Milk	Fat	Protein	Fat	Protein	
1	429,201	0^a^	0^a^	0^a^	0^a^	0^a^	0^a^
2	423,085	6.3 (2.17)^b^	1.4 (0.09)^b^	0.2 (0.07)^bc^	0.02 (0.001)^b^	−0.002 (0.0005)^b^	0.006 (0.0022)^b^
3	317,985	9.9 (2.35)^bc^	1.4 (0.09)^b^	0.3 (0.08)^b^	0.02 (0.001)^b^	0.000 (0.0006)^a^	−0.005 (0.0023)^c^
4	239,098	15.7 (2.56)^cd^	1.2 (0.10)^bd^	0.4 (0.08)^b^	0.01 (0.001)^b^	0.000 (0.0006)^ad^	−0.011 (0.0025)^cd^
5	169,980	16.1 (2.86)^c^	1.4 (0.11)^b^	0.4 (0.09)^b^	0.02 (0.002)^b^	0.002 (0.0007)^cd^	−0.012 (0.0029)^de^
6	111,244	14.2 (3.34)^c^	1.1 (0.13)^bc^	0.3 (0.11)^b^	0.02 (0.002)^b^	0.002 (0.0008)^ce^	−0.013 (0.0033)^de^
7	68,578	19.8 (4.06)^d^	1.0 (0.16)^cd^	0.4 (0.13)^b^	0.01 (0.002)^b^	0.001 (0.0010)^ac^	−0.016 (0.0040)^de^
8	38,262	9.7 (5.23)abcd	0.7 (0.21)^c^	0.2 (0.17)^ab^	0.02 (0.003)^b^	0.005 (0.0013)^e^	−0.013 (0.0052)cde
9	20,120	13.0 (7.06)abcd	0.5 (0.28)^c^	0.1 (0.23)^ab^	0.02 (0.004)^b^	0.004 (0.0017)^ce^	−0.027 (0.0070)^e^
10	16,322	7.1 (7.88)abcd	−0.3 (0.32)^a^	−0.2 (0.26)^ac^	0.01 (0.004)^b^	0.005 (0.0019)^e^	−0.027 (0.0078)^de^

a–e Different superscripts within columns denote differences (*P* < 0.05) between solutions without adjustment for multiple testing.

**Table 2:** Number of records (N), along with yields and composition (including SCS), relative to first parity cows (SE in parentheses), for cows of different parities

Parity	N	Yield (kg)			Composition (%)		SCS (log_e_ units)
		Milk	Fat	Protein	Fat	Protein	
1	446,202	0	0	0	0	0	0
2	381,007	1,022 (1.3)	39.4 (0.06)	39.3 (0.04)	−0.07 (0.001)	0.04 (0.0003)	−0.16 (0.002)
3	303,344	1,481 (1.5)	59.7 (0.07)	56.4 (0.05)	−0.06 (0.001)	0.05 (0.0003)	−0.03 (0.002)
4	237,499	1,725 (1.7)	69.4 (0.07)	64.3 (0.06)	−0.06 (0.001)	0.04 (0.0004)	0.08 (0.002)
5	177,110	1,822 (1.9)	72.8 (0.09)	66.8 (0.07)	−0.07 (0.001)	0.03 (0.0004)	0.21 (0.002)
6	124,532	1,805 (2.3)	71.2 (0.1)	65.1 (0.08)	−0.08 (0.001)	0.01 (0.0005)	0.34 (0.003)
7	78,320	1,735 (2.7)	68.2 (0.12)	61.9 (0.10)	−0.08 (0.001)	0.01 (0.0006)	0.47 (0.003)
8	45,539	1,606 (3.4)	62.8 (0.15)	56.7 (0.12)	−0.07 (0.002)	0.002 (0.0008)	0.61 (0.004)
9	23,770	1,446 (4.5)	56.1 (0.20)	50.4 (0.16)	−0.07 (0.002)	−0.004 (0.001)	0.74 (0.005)
10	16,552	1,177 (5.8)	45.2 (0.25)	40.4 (0.20)	−0.06 (0.003)	−0.01 (0.0013)	0.90 (0.007)

The model regression coefficients (SE) of the yield trait on its respective estimated breeding value was 1.05 (0.003), 0.89 (0.0036), and 0.98 (0.004) for milk, fat, and protein yield, respectively, which is close, yet different (*P* < 0.001) to the expectation of 1 and similar to that reported previously in a validation of genetic evaluations in Irish dairy cows (Ring et al., 2021); the respective model regression coefficients for milk fat and protein percentage were 1.11 (0.003) and 1.17 (0.002), whereas that for SCS was 1.16 (0.008), all of which differed (*P* < 0.001) from 1. Relative to no recorded assistance at calving, the requirement for slight assistance, severe assistance and veterinary assistance resulted in, on average, 20.1 kg (SE = 5.6 kg), 68.6 kg (SE = 7.16 kg), and 188.9 kg (SE = 12.5 kg) less 305-d milk yield, respectively. The association between greater required assistance at calving and reduced subsequent milk yield has been well documented in other dairy cow populations (Berry et al., 2007). The 100% heterosis effects associated with an F_1_ cross was associated with 169.5 kg (SE = 14.25 kg), 7.82 kg (SE = 0.566 kg), 7.17 kg (SE = 0.467 kg) more milk, fat, and protein yield, respectively.

Both dam parity and cow parity were associated (*P* < 0.001) with all 6 performance traits explored. The model dam parity solutions for the 6 traits explored are in Table 1. Mean milk yield per cow increased slightly and at a diminishing rate with increasing dam parity number from parity 1 (i.e., pregnant as nulliparous heifers) to parity 7 after which it declined again with advancing parity number; in fact, dam parity milk yield effects after parity 4 did not differ from each other. A similar parity trend existed for protein yield increasing with dam parity up to parity 5 after which it declined albeit many of the parity effects in older parity dams did not differ from each other. For fat yield, cows born to parity 1 dams yielded less than contemporaries born to parity 2 dams with dam parity having little effect thereafter until parity 5 where a reduction thereafter in mean progeny fat yield was detected. Nonetheless, relative to the mean performance of a first parity cow, the difference between the extreme dam parity effects was <0.6% of the mean yield of first parity cows. The mean milk protein percent was least in progeny from parity 2 dams increasing thereafter while the mean milk fat percent for progeny of parity 2 to 10+ dams were similar, all of which were, on average, higher than progeny from parity 1 dams (i.e., those pregnant as nulliparous heifers). Although the mean lactation SCS of cows from parity 2 dams was higher than those from parity 1 dams, with each advancing dam parity number thereafter, the mean lactation SCS of the cow progeny reduced albeit many of the parity effects did not differ from each other.

Age at calving of the dam relative to the mean per parity was also associated (*P* < 0.001) with all 6 traits explored. Considering dams calving within 4 mo of the median calving age per parity (age effects more than 4 mo were associated with large standard errors), progeny from dams who calved younger than the median produced more milk, milk fat, and milk protein with the opposite being true for those calving older than the parity median. Progeny from dams that calved 4 mo younger than the median produced, on average, 46.48 (SE = 17.81), 2.46 (SE = 0.71), and 1.95 kg (SE = 0.58) more milk, milk fat, and milk protein over the 305 d of lactation than those calving at the median age.

The model cow parity solutions for the 6 performance traits explored are in Table 2. Mean milk, fat, and protein yield increased at a decreasing rate with advancing parity up to the fifth parity, after which it declined at an accelerating rate; the mean 305-d lactation yield of a fifth parity cow was, on average, 30% to 33% greater than that of a first parity cow. Mean milk protein percent increased with cow parity number to a peak at parity 3 after which it consistently reduced with advancing parity. Milk fat percent of parity 2 to parity 10+ cows was relatively similar, all of which were lower than the mean fat percent of parity 1 cows. Mean lactation SCS reduced from cow parity 1 to parity 2, after which it increased almost linearly with each incremental increase in cow parity number.

The justification for exploring the dam parity effects in the present study arose from the tendency among some dairy producers to use sexed sorted semen proportionally more on the younger animals in their herd. Hence, quantifying if this has any repercussions on subsequent progeny performance requires exploration. The proportion of females calves in the present study recorded to be born to first (i.e., pregnant as nulliparous), second, and third parity cows calving in the year 2024 was 59%, 54%, and 53%, respectively, with the weighted mean for older parity cows being 51%; hence, based on the data used in the present study at least, sexed semen appears to be used proportionally more in younger cows and especially heifers. Calves from first parity dairy cows (i.e., pregnant as nulliparous heifers) tend to be born lighter than those from older parity cows (Hickson et al., 2015; Handcock et al., 2021; Condon et al., 2024) with this effect holding true also when calving themselves for the first time (Heinrichs et al., 2005). The association between lighter dairy cows at first calving and the production of less milk relative to their heavier contemporaries has previously been demonstrated (Carson et al., 2002). Hence, the expectation could be that the lactation performance of cows from younger parity dams should be less than that from older parity dams. This hypothesis held true in the present study but is largely in direct contrast to that documented in other dairy cow populations where lower lactation yield was associated with cows born to older parity dams (Storli et al., 2014; Van Eetvelde et al., 2020; Handcock et al., 2021). Irrespective of the direction of the association between dam parity number and cow yield across the different studies, the documented dam parity effect sizes were all small representing <3.5% of the mean yield of the population (present study; Van Eetvelde et al., 2020; Handcock et al., 2021). In the present study, the cow progeny from a seventh parity dam is expected to yield just 19.8 kg more milk over a 305-d lactation than progeny from first parity dams, which represents less than 0.5% of the mean first parity yield; the respective dam parity effect was 0.06% and 0.02% for fat yield and protein yield, respectively, in the present study. The 19.8 kg extra milk over a 305-d lactation in progeny from older parity dams is not even a single day's milk yield based on the mean mature cow 305-d yield in the present study. Limiting the association analyses to a dataset of 1,415,489 records from cows that were at least 90% Holstein-Friesian did not affect the results.

Cow reproductive performance is improving in many dairy cow populations (Berry et al., 2014; García-Ruiz et al., 2016). Hence, involuntary culling due to infertility is reducing, creating an opportunity to increase cow longevity. The benefit of increasing cow longevity relative to the opportunity costs of same requires information on cow parity effects on productivity. Heretofore, the generally poor reproductive performance in many dairy cow populations has meant that having sufficient records to estimate older parity effects was not possible—furthermore, many studies truncate their data to the first 3 or 5 parities, or collapse parities greater than 5 into a single group. The present study included parities up to parity 15; only 0.1% of the data were from parity 11 or greater so these were all collapsed with parity 10. Despite mean yield per parity reducing after parity 5, parity 10+ cows still yielded 19% to 21% more milk, fat, and protein than first parity cows. Although progeny from these older parity cows are expected to yield (slightly) more, genetically these older parity cows are expected to, on average, be inferior on total merit index so should not be considered as candidate parents of the next generation of dairy herd replacements—they would, however, be likely strong candidates for beef-on-dairy matings. In the present study, less than 1% of cows calving for the first time in the year 2024 were from parity 10+ dams; 22% were from nulliparous heifers.

In conclusion, cow lactation milk yield increased as the parity number of their dam increased up to a parity number of 7 after which it declined; for fat and protein yield, the peak yield occurred at earlier dam parities. Nonetheless, the biological significance of this dam parity effect was minimal. Although the mean SCS of cows was greatest from parity 2 dams, the mean lactation SCS declined thereafter as dam parity number increased. Mean yield increased with cow parity up to parity 5 after which it declined; mean milk, fat, and protein yield for fifth parity cows was 30% to 33% higher than first parity contemporaries. Somatic cell score was lowest for second parity cows, increasing almost linearly thereafter with parity number.
